# Relationship of urinary bisphenol A in childhood on thyroid hormone function in adolescents: a cohort study

**DOI:** 10.1371/journal.pone.0322658

**Published:** 2025-05-22

**Authors:** Jung Eun Choi, Eun Jeong Choi, Seonhwa Lee, Bohyun Park, Hye Ah Lee, Young Sun Hong, Eunhee Ha, Hae Soon Kim, Hyesook Park

**Affiliations:** 1 Department of Pediatrics, College of Medicine, Ewha Womans University, Seoul Hospital, Seoul, Republic of Korea; 2 Department of Preventive Medicine, College of Medicine, Ewha Womans University, Seoul, Korea; 3 National Cancer Control Institute, National Cancer Center, Goyang, Korea; 4 Clinical Trial Center, Ewha Womans University Mokdong Hospital, Seoul, Republic of Korea; 5 Department of Internal Medicine, College of Medicine, Ewha Womans University, Seoul, Republic of Korea; 6 Department of Occupational and Environmental Medicine, College of Medicine, Ewha Womans University, Seoul, Korea; 7 Graduate Program in System Health Science and Engineering, Ewha Womans University, Seoul, Republic of Korea; King Faisal Specialist Hospital and Research Center, SAUDI ARABIA

## Abstract

**Background:**

Bisphenol A (BPA) is a type of endocrine-disrupting chemical utilized in the production of plastics like epoxy resins and polycarbonate polymers. BPA exhibits weak estrogenic and potent anti-androgenic effects, and prior research has linked it to disturbances in thyroid function. This study aims to assess the potential association between early childhood exposure to urinary bisphenol A and thyroid hormone levels in pubertal children from Korea.

**Methods:**

Participants were drawn from the Ewha Birth and Growth Cohort Study, encompassing individuals who visited Ewha Women’s Mokdong Hospital between 2001 and 2005. The concentration of urinary BPA was repeatedly measured for each subject at ages 3–5 years and 7–9 years. Serum levels of free triiothyronine (T3), free thyroxine (T4), and thyroid-stimulating hormone (TSH) were measured at ages 10–12 years in a subgroup of 128 out of 164 subjects who had undergone repeated BPA concentration measurements. We utilized the SAS program to analyze possible links between childhood exposure to BPA and thyroid hormone function in adolescence. Additionally, we explored how exposure to BPA during two specific periods influenced changes in thyroid hormone levels.

**Results:**

The study observed that urinary BPA levels at ages 3–5 years were not notably linked to thyroid hormone levels in adolescents aged 10–12 years. However, BPA levels at ages 7–9 years were significantly associated with free T3 levels in girls aged 10–12 years. Conversely, exposure to BPA did not result in significant differences in thyroid hormone levels among boys. The study did not find statistically significant connections between levels of urinary BPA and the other thyroid hormones, specifically TSH and free T4. There was a significant decrease in the concentration of free T3 in girls with higher BPA concentrations.

**Conclusions:**

BPA exposure in childhood affects thyroid function in adolescent girls. This relationship may contribute to an increased prevalence of thyroid disorders in adolescents due to environmental influences.

## Introduction

Bisphenol A (BPA) is an endocrine-disrupting chemical and is mainly used in plastic, epoxy resins, and food can lining, making it a pervasive environmental contaminant. Found in food containers, water bottles, and thermal receipts BPA is detectable in the biological samples of nearly all individuals worldwide. as a synthetic resin and coating material inside cans for food storage [1]. BPA is easily transferred to water, food, and air and accidentally transmitted to humans through ingestion, inhalation, or skin contact [2]. Despite the knowledge that BPA is harmful, it is still widely used, and its substitutes, such as bisphenol F (BPF) and bisphenol S (BPS), cannot be considered completely free of adverse effects. Due to their structural similarities to BPA, these bisphenols may also disrupt thyroid function [1].

BPA has been reported to interfere with hormonal functions, particularly the actions of estrogen and thyroid hormones at various levels [3]. Several in vitro and animal studies have found evidence of an association between BPA exposure and the disruption of thyroid hormone through multiple mechanisms [4,5,6]. These mechanisms include the inhibition of the sodium/iodide symporter (NIS), competitive binding to thyroid hormone transport proteins, and alterations in thyroid hormone receptor (THR) gene expression [7]. Additionally, bisphenol’s analogues have been found to induce androgenic and estrogenic effects, contributing to broader endocrine disruption [4].

Recently, several epidemiological studies have reported an association of BPA exposure with thyroid hormone levels. However, the results are still controversial, and studies on children are particularly limited [2]. Some studies have found that prenatal BPA exposure is associated to reduce TSH and increased total tri-iodothyronine (TT3) levels in female offspring, while others suggest that BPA exposure in boys may decrease TT3 levels [8,9]. Other study has reported varying trends in free T3 and free T4 levels depending on the cumulative exposure to bisphenol mixtures. Some research indicates that exposure to BPA analogues, such as BPF and BPS, may also influence thyroid hormone levels, with mixed findings on whether they increase or decrease specific thyroid parameters [10,11]. These inconsistencies may be due to differences in study populations, exposure levels, and measurement methodologies, emphasizing the need for further research to clarify the relationship between bisphenol exposure and thyroid function.

Children are more vulnerable than adults to environmental chemicals because they have a small body surface per weight. In addition, children do not have fully developed nervous, immune, reproductive and digestive systems and have difficulty controlling hazardous environments. The puberty period is a critical window of susceptibility to endocrine-disrupting chemicals (EDCs) including BPA, may interfere with hormonal regulation, potentially disrupting pubertal timing and contributing to developmental abnormalities [12]. Adequate thyroid hormone is essential for optimal development of children, especially in brain development and intelligence before the age of three, as well as sexual development and reproductive function [5]. EDCs exposure, such as BPA, in childhood can cause hormone disturbance, bring about various health problems, and have long-term effects on health in adult life [3]. Moreover, younger children had a higher BPA concentration compared to older people [13]. Therefore, it is necessary to study the endocrinologic effect of BPA on thyroid function in adolescence, which plays a hormone-disrupting role during childhood.

This study aims to investigate the relationship between urinary bisphenol A exposure in childhood and the thyroid hormone of pubertal children in Korea.

## Methods

### Study population

The subjects of this study were composed of the prospective birth cohort of the Ewha Birth and Growth Cohort Study, established at Mokdong Hospital between 2001 and 2005. The first follow-up started when subjects were 3 years of age and the follow-ups was performed every year after 2005. Details of the cohort composition and methodology are described in a previous study [14]. As a result of the analysis of the demographic characteristic, there was no difference between the two groups that the included subjects and non-included subjects in this study. Therefore, the possibility of selection bias may be low, and this has also been reported in previous studies that included the same subjects [15,16].

Of the 940 children enrolled in the birth cohort,164 subjects who participated in both the early childhood (at ages 3–5) and early school-age (at ages 7–9) follow-ups and provided repeat BPA measurements from urine samples were selected. Finally, 128 of 164 children who participated in the follow-up at 10–12 years of age and had no missing values were included in the analyses.

All subjects and their parents or guardians agreed to participate in this study through informed consent. The Institutional Review Board on Human Subjects at Ewha Womans University approved the study protocol (SEUMC- 2019-04-035).

### Exposure assessment

The concentration of urinary bisphenol A was measured repeatedly for the same subjects at 3–5 years old and 7–9 years old Urinary bisphenol A concentrations in the subjects were determined by high-performance liquid chromatography method coupled with tandem mass spectrometry featuring Agilent 1200 series and Agilent 6410 Triple Quad liquid chromato-graphy/mass spectrometry platforms (Agilent Technologies Inc., Santa Clara, CA). The limit of detection (LOD) was 0.05 μg/L, and the accuracy of the analysis and coefficient of variation (CV) were 100.05% and 1.67%, respectively. Creatinine was measured using the Jaffé method with the OSR6178 or OSR6678 reagent and an Olympus AU 680 spectrometer (Beckman Coulter, USA). We log-transformed BPA in the analysis to normalize its distribution. To compare exposures among groups, subjects were classified into tertiles based on urinary BPA concentrations at baseline. In addition, a dose-response relationship of BPA exposure with thyroid function was explored via trend analysis after the subjects were divided into tertiles based on BPA exposure concentration.

### Outcome assessment

Serum free T3, free T4 and TSH levels were measured in the thyroid function test. The analysis was performed by electrochemiluminescence immunoassay (Elecsys FT3 III, Elecsys FT4 II; Roche, Germany). TSH was measured using an immunoassay (E411).

### Covariate

Through a review of the literature, sex, age, mother’s education, parents smoking and current body mass index (BMI). The information for covariates was collected from the questionnaires. Current BMI was calculated using body measurements obtained during follow-up as weight (kg) divided by the square of height (m^2^). Height and weight were measured in 0.1-cm and 0.1-kg units using an automatic height and weight measurement system (DS-102; Dong Sahn Jenix Co., Ltd., Seoul, Korea), without shoes and in light clothing. Maternal educational levels (elementary/middle school, high school, college, graduate school or higher) were divided into two categories (Graduated from high school or Some college or higher). A questionnaire item was used to assess each child’s exposure to secondhand smoke on follow-up at the age of 10–12 years.

### Statistical analysis

In the descriptive analysis, continuous variables are presented as the mean and standard deviation when the normal distribution is satisfied and presented as the median with interquartile ranges when they are not distributed normally. Categorical data are presented as the frequency with percentage.

Simple linear regression analysis was used to examine the associations of urinary BPA concentrations measured at two time points (3–5 years and 7–9 years), and serum FT3, FT4, and TSH concentrations at ages 10–12. Considering a normal distribution, urinary BPA concentrations were log-transformed and used in the analysis. Multivariate linear regression analysis was performed, adjusting for sex, age, maternal education level, secondhand smoke, and current BMI.

To elucidate mean differences in thyroid hormone concentrations at ages 10–12 according to BPA exposure, participants were categorized into tertiles based on urinary BPA concentrations measured at two time points, and generalized linear models (GLM) and trend analyses were conducted.

Additionally, the interaction effect of urinary BPA exposure levels (tertiles) at two time points on thyroid hormone concentrations at ages 10–12 was examined using GLM.

Statistical analysis was performed using the SAS program version 9.4 (Statistical Analysis System version 9.4, SAS Institute, Cary, NC, USA), and statistical significance in the two-tailed test was determined as a *p*-value < 0.05

## Results

The basic characteristics of the subjects are presented in Table 1. The study population included a total of 128 subjects, 61 boys and 67 girls. The mean age of the participants was 10.1 years. The mean BMI for boys was slightly higher than for girls. Most children in this study were not exposed to secondhand smoke. In the case of thyroid function, the mean levels of TSH, free T4 and free T3 among children were 2.95 ± 1.37 μU/mL, 1.48 ± 0.20 ng/mL, 4.59 ± 0.46 ng/mL. TSH and free T4 were present in statistically higher concentrations in boys than girls (*p < 0.05*). However, there was no statistical difference in the free T3 level. The urinary BPA concentration was higher in boys (0.78 µg/g creatinine) than in girls aged 3–5 years (0.60 µg/g creatinine; *p = 0.06*), but no obvious difference was seen in adolescents aged 7–9 years.

**Table 1:** Characteristics of study subjects.

	Total		Boys		Girls		*p*
**Bisphenol A**							
3-5 years	128	0.78 (0.39-1.45)	61	0.78 (0.39-1.45)	67	0.60 (0.34-1.12)	0.06
7-9 years	127	0.62 (0.38-1.17)	61	0.62 (0.38-1.17)	66	0.58 (0.36-1.07)	0.62
**Thyroid function at 10–12years**							
TSH (ulU/mL)	128	2.76 (1.95-3.67)	61	3.02 (2.34-4.17)	67	2.53 (1.72-3.54)	0.01
FreeT4 (ng/dL)	128	1.46 (1.36-1.56)	61	1.49 (1.39-1.6)	67	1.42 (1.33-1.52)	0.03
Free T3 (ng/dL)	128	4.59 ± 0.46	61	4.62 ± 0.42	67	4.57 ± 0.5	0.64
							
**Covariate variable**							
Age(years)	128	10.12 ± 0.35	61	10.13 ± 0.39	67	10.1 ± 0.31	0.67
BMI(kg/m^2^)	128	18.14 ± 2.98	61	18.66 ± 3.12	67	17.66 ± 2.78	0.06
Secondhand Smoke	46 (36.22%)		21 (35.00%)		25 (37.31%)		0.79
Mother’s education level							
Graduated from high school	28		8 (13.11%)		20 (29.85%)		0.02
Some college or higher	100		53 (86.89%)		47 (70.15%)		

Abbreviation: TSH, thyroid stimulating hormone; Free T4, Free Thyroxine; Free T3, Free Tri-iodothyronine; BMI, body mass index.

Values are presented as mean ± SD, median (interquartile range), and frequency (percentage).

Table 2 shows the association between log-transformed urinary BPA levels in ages 3–5 years and 7–9 years and the serum thyroid hormone at 10–12 years. The relationship between BPA at age 7–9 and free T3 was significant for girls, both in the crude model and adjusted model by sex, age, mother’s education, parent smoking status, and current BMI (Coefficients between BPA at age 7–9 and free T3 were -0.14 (-0.26, -0.02) and -0.15 (-0.28, -0.03), -0.15 (-0.28, -0.03)). Higher urinary BPA concentrations in girls aged 7–9 years were significantly associated with a lower free T3 level. There were no significant differences in the levels of thyroid hormone by the urinary BPA level in both boys and girls aged 3–5 year.

**Table 2:** Linear regression analysis results for the effect of log-transformed urinary BPA concentrations at two time points on serum thyroid concentrations at ages 10–12.

Total			TSH (ulU/mL)		FT4 (ng/dL)		FT3 (ng/dL)	
*β* (95%CI)	*p*	*β* (95%CI)	*p*	*β* (95%CI)	*p*
Crude	Bisphenol A at 3–5 years	Total	0.07 (-0.18, 0.33)	0.56	0.03 (-0.00, 0.07)	0.07	0.06 (-0.03, 0.14)	0.20
Model		Boys	0.05 (-0.31, 0.41)	0.80	0.01 (-0.05, 0.06)	0.85	0.04 (-0.08, 0.15)	0.53
		Girls	-0.04 (-0.41, 0.34)	0.85	0.05 (-0.00, 0.1)	0.07	0.07 (-0.07, 0.21)	0.30
	Bisphenol A at 7–9 years	Total	0.09 (-0.17, 0.34)	0.51	0.00 (-0.04, 0.04)	0.98	-0.09 (-0.18, -0.01)	0.04
		Boys	-0.00 (-0.40, 0.39)	0.98	0.02 (-0.04, 0.08)	0.56	-0.03 (-0.16, 0.09)	0.61
		Girls	0.12 (-0.21, 0.46)	0.46	-0.02 (-0.06, 0.03)	0.53	-0.14 (-0.26, -0.02)	0.03
Adjusted	Bisphenol A at 3–5 years	Total	0.08 (-0.19, 0.35)	0.55	0.03 (-0.01, 0.07)	0.13	0.07 (-0.03, 0.16)	0.16
Model 1		Boys	0.20 (-0.19, 0.59)	0.32	0.02 (-0.04, 0.08)	0.55	0.09 (-0.04, 0.21)	0.17
		Girls	0.06 (-0.32, 0.44)	0.74	0.05 (-0.01, 0.1)	0.08	0.04 (-0.10, 0.18)	0.60
	Bisphenol A at 7–9 years	Total	0.09 (-0.16, 0.34)	0.46	-0.00 (-0.04, 0.04)	0.92	-0.10 (-0.19, -0.01)	0.03
		Boys	0.04 (-0.35, 0.43)	0.82	0.02 (-0.04, 0.08)	0.50	-0.02 (-0.15, 0.10)	0.73
		Girls	0.18 (-0.16, 0.51)	0.30	-0.02 (-0.06, 0.03)	0.53	-0.15 (-0.28, -0.03)	0.01
Adjusted	Bisphenol A at 3–5 years	Total	0.09 (-0.17, 0.34)	0.51	0.03 (-0.01, 0.07)	0.13	0.07 (-0.03, 0.16)	0.15
Model 2		Boys	0.22 (-0.16, 0.60)	0.25	0.02 (-0.05, 0.08)	0.61	0.09 (-0.03, 0.22)	0.13
		Girls	0.05 (-0.33, 0.42)	0.81	0.05 (-0.00, 0.1)	0.06	0.04 (-0.11, 0.18)	0.62
	Bisphenol A at 7–9 years	Total	0.10 (-0.15, 0.34)	0.44	-0.00 (-0.04, 0.03)	0.91	-0.10 (-0.18, -0.01)	0.03
		Boys	0.04 (-0.34, 0.43)	0.83	0.02 (-0.04, 0.08)	0.50	-0.02 (-0.15, 0.10)	0.71
		Girls	0.18 (-0.14, 0.51)	0.27	-0.02 (-0.06, 0.03)	0.50	-0.15 (-0.28, -0.03)	0.01

Abbreviations: 95% CI, 95% confidence intervals; TSH, thyroid stimulating hormone; Free T4, Free Thyroxine; Free T3, Free Triiodothyronine.

Adjusted Model 1 adjusted for sex, age, mother’s education level, and secondhand smoke.

Adjusted Model 2 adjusted for sex, age, mother’s education level, secondhand smoke, and current BMI.

Table 3 show the relationship between urinary BPA tertiles and serum thyroid hormone levels. To investigate the possible dose-response relationship of BPA exposure and thyroid hormone function, the mean values and trends were calculated after dividing the BPA exposure levels into tertiles.

**Table 3:** Association between urinary BPA exposure levels in childhood and serum thyroid hormone concentrations in adolescence.

		TSH (ulU/mL)		Free T4 (ng/dL)		Free T3 (ng/dL)	
		LS Mean (95% CI)	*p*(*p* for trend)	LS Mean (95% CI)	*p* (*p* for trend)	LS Mean (95% CI)	*p* (*p* for trend)
Bisphenol A at 3–5 years							
Total	T1	2.26 (1.32, 3.21)	0.89 (0.75)	1.44 (1.29, 1.58)	0.83 (0.59)	4.62 (4.28, 4.96)	0.27 (0.31)
	T2	2.14 (1.14, 3.15)		1.45 (1.30, 1.6)		4.55 (4.19, 4.91)	
	T3	2.14 (1.15, 3.12)		1.46 (1.31, 1.61)		4.72 (4.37, 5.07)	
Boys	T1	2.25 (1.09, 3.41)	0.31 (0.57)	1.53 (1.34, 1.72)	0.78 (0.63)	4.55 (4.18, 4.93)	0.18 (0.07)
	T2	2.92 (1.72, 4.13)		1.48 (1.28, 1.67)		4.62 (4.23, 5.01)	
	T3	2.49 (1.38, 3.60)		1.49 (1.30, 1.67)		4.81 (4.45, 5.17)	
Girls	T1	2.56 (1.90, 3.21)	0.09 (0.59)	1.36 (1.26, 1.45)	0.31 (0.15)	4.68 (4.42, 4.94)	0.8 (0.94)
	T2	1.77 (1.05, 2.48)		1.42 (1.32, 1.53)		4.59 (4.30, 4.88)	
	T3	2.46 (1.77, 3.16)		1.43 (1.33, 1.54)		4.68 (4.41, 4.96)	
Bisphenol A at 7–9 years							
Total	T1	1.90 (0.92, 2.89)	0.3 (0.23)	1.46 (1.31, 1.61)	0.5 (0.31)	4.77 (4.42, 5.12)	0.05 (0.01)
	T2	2.33 (1.39, 3.28)		1.46 (1.32, 1.60)		4.64 (4.30, 4.98)	
	T3	2.23 (1.25, 3.21)		1.41 (1.26, 1.56)		4.52 (4.17, 4.87)	
Boys	T1	2.03 (0.85, 3.22)	0.11 (0.82)	1.49 (1.29, 1.69)	0.69 (0.72)	4.71 (4.31, 5.11)	0.45 (0.35)
	T2	2.88 (1.79, 3.97)		1.53 (1.35, 1.71)		4.76 (4.39, 5.12)	
	T3	2.13 (1.02, 3.23)		1.47 (1.28, 1.66)		4.59 (4.21, 4.96)	
Girls	T1	2.04 (1.37, 2.70)	0.27 (0.12)	1.43 (1.33, 1.52)	0.48 (0.28)	4.83 (4.58, 5.08)	0.06 (0.02)
	T2	2.21 (1.44, 2.98)		1.42 (1.31, 1.53)		4.63 (4.33, 4.92)	
	T3	2.65 (1.96, 3.35)		1.36 (1.26, 1.46)		4.48 (4.22, 4.74)	

Abbreviations: LS means, Least-squares means; 95% CI, 95% confidence intervals; TSH, thyroid stimulating hormone; Free T4, Free Thyroxine; Free T3, Free Triiodothyronine.

Adjusted for sex, age, mother’s education level, secondhand smoke, and current BMI.

Bisphenol A (µg/g creatinine) at 3–5 years: 1st tertile (T1)<0.48, 2nd tertile (T2) 0.48–1.03, 3rd tertile (T3)>1.03. Bisphenol A (µg/g creatinine) at 7–9 years: 1st tertile (T1)<0.41, 2nd tertile (T2) 0.41–0.99, 3rd tertile (T3)>0.99.

In early school age, 7–9 years old, the free T3 level decreased significantly as the concentration of BPA increased (T1: 4.77, T2: 4.64, T3: 4.52, *p for trend = 0.01*), and the dose-dependent relationships were more evident in girls than in boys (boys, T1: 4.71, T2: 4.76, T3: 4.59, *p* for trend = 0.35; Girls, T1: 4.83, T2: 4.63, T3: 4.48, *p* for trend = 0.02). At age 3–5, early childhood, thyroid hormone had no significant association with an increase in the BPA concentration (*p for trend = 0.31*).

**Fig 1:**
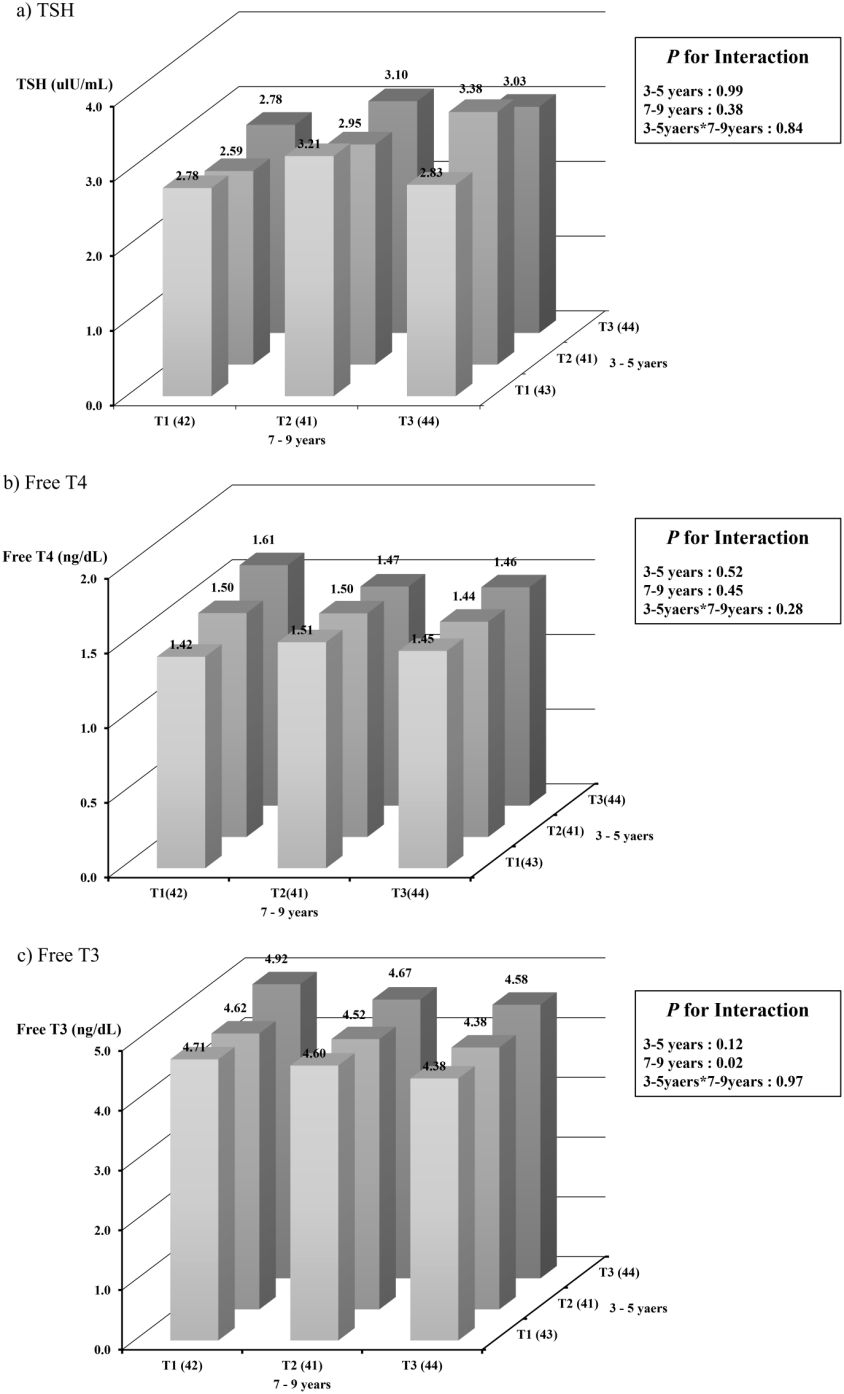
The interaction effect of urinary bisphenol A exposure levels at two time points during childhood on serum thyroid hormone concentrations in adolescence. (a) The interactions between TSH at 3–5years and TSH at 7–9years (*p* of 3–5 years = 0.99, 7–9 years = 0.38, Interaction = 0.84.); (b) The interactions between Free T4 at 3–5years and Free T4 at 7–9years (*p* of 3–5 years = 0.52, 7–9 years = 0.45, Interaction = 0.28.); (c) The interactions between Free T3 at 3–5years and Free T3 at 7–9years (*p* of 3–5 years = 0.12, 7–9 years = 0.02, Interaction = 0.97).

To identify interaction effects in addition to independent effects at the two time points, we evaluated the interactive effects of urinary BPA levels at two time points in childhood on serum thyroid hormone concentrations in adolescence using GLM. Our results are presented in Figure 1. We did not find any significant interaction between the two periods of early childhood and early school-age. However, we found that the comparison of FT3 level according to the BPA tertiles (T1 lowest, T2 middle, T3 highest) at age 7–9 years provided statistically significant results (*p = 0.02*).

## Discussion

In this study, we evaluated the effects of BPA exposure in childhood on thyroid hormone function around puberty, at 10–12 years old. The BPA level at 3–5 years had no significant relationship with thyroid hormone at 10–12 years. As the BPA exposure increased at 7–9 years, the free T3 level of girls aged 10–12 years significantly decreased. The higher the concentration of BPA in school-age children of 7–9 years, the larger the adverse effect on thyroid hormone in girls. Our data indicates that there were no significant effects of BPA exposure in early childhood on later health, but exposure at an early school age had a significant effect on thyroid hormone. It could be said that the period of 7–9 years of age had a larger impact on thyroid hormone because the children were exposed close to the time of the testing. These results show that exposure to BPA close to puberty can affect thyroid hormone in adolescence.

In a previous study, the association of prenatal BPA and early childhood BPA, BPF, and BPS exposure with thyroid function in 6-year-old children was investigated [3]. In this study, as the concentration of bisphenol F increased, the free T4 level of girls decreased. In addition, a large cohort study of 6003 adults through the Korean National Environmental Health Survey found that the higher the BPA exposure concentration, the lower the TSH level [17]. In China’s prospective cohort study, maternal exposure to BPA affected fetal thyroid hormone, significantly reduced T3 level in cord blood, and increased behavioral problems, as it affected neurobehavioral development between ages 2 and 4 [18]. The results of this study also showed that TSH increased, free T4 decreased, and free T3 decreased significantly according to the exposure amount in ages 7–9, which were similar results to previous studies.

A large cohort study using data from the Korean National Environmental Health Survey examined the relationship between BPA exposure and thyroid function by BMI. This study found that BPA was negatively associated with T3 and T4 levels in adults with higher BMI, but no obvious association was observed between BPA and TSH [19]. Another study investigated the influence of BPA on thyroid volume and structure independent of iodine levels in school children and found that higher urinary BPA concentrations were inversely associated with thyroid volume and the risk of multiple nodules. This suggests that BPA exposure may contribute to alterations in thyroid morphology and function even when iodine intake is sufficient [20]. A recent meta-analysis of 11 cohort studies involving 5,363 children found that prenatal bisphenol exposure had sex-differentiated effects on thyroid function. Notably, both the meta-analysis and our cohort study observed an association between bisphenol and decreased free T3 levels in children, reinforcing the impact of endocrine disruption during critical developmental period. These findings emphasize the need for stricter regulations on BPA and its analogs to mitigate potential thyroid hormone disruptions [8].

Several in vitro and animal studies have provided evidence for an association between BPA exposure and the disruption of thyroid hormone [4,5,6]. Bisphenols can disrupt thyroid hormone signaling and action by altering the transcription of thyroid function-related genes such as TSH-ß, TRs and deiodinase. It is likely that BPA alters the relationship of the TR to various co-modulatory molecules. Specifically, BPA can antagonize T3 action at the transcriptional level, which suggests that BPA and analogues of BPA derivatives interfere with thyroid hormone signaling during development [21]. The biological mechanism through which BPA and thyroid hormone function are related is currently ill-defined and warrants further detailed investigation [5,22].

Despite previous studies with subjects of various ages and both sexes, such as early childhood, adults and pregnant females, our study differs from other studies in that it investigates thyroid hormone function in adolescence [23,24,25,26,27]. During puberty, thyroid function changes and thyroid volume increases by adaptation to sexual development. It is not yet clear how much thyroid hormone changes due to certain puberty stages or specific pubertal development patterns [28]. In addition, in this study, thyroid hormone dysfunction was more pronounced in girls, which is consistent with previous findings on 6-year-old children [2]. In boys, no significant differences in thyroid hormone levels were observed by exposure to BPA. These results show that BPA affects thyroid hormones differently according to sex. Thyroid disease has a higher prevalence among females than males in adulthood, and epidemiologic reports have revealed the potential mechanisms of estrogen on thyroid function and growth regulation [12]. A previous study revealed that BPA clearance was faster in females than in males [29]. Another study also reported a sex difference in serum BPA levels due to differences in androgen-associated BPA metabolism [30]. An animal study suggested that sex differences may be associated with sex hormone-related markers because BPA functions as an estrogen analogue [31].

Free T3, instead of total T3, was measured in this study. The free portion of T3 is considered more relevant to the biological action than total T3 [32]. Another strength of our study is that birth cohort data were used to prospectively assess causal relationships between BPA exposure and the thyroid function test. Our results were significant even after adjustment for various covariates. Another strength is that our study measured BPA exposure at two points and evaluated the effects on subjects at the same time.

There are several limitations to our study. First, BPA exposure was measured with a single spot urine sample, which may not represent the average long-term exposure due to short half-life and body variability of bisphenols [15,33]. Second, this study included a limited number of bisphenols. Although BPA is one of the most well-known raw materials for production of plastics and epoxy resin, BPF and BPS have also recently been reported to have toxic effects on reproduction, development, and aging. Third, the relatively small sample size of the study may lower the accuracy of our results. Fourth, one potential confounding factor that was not controlled for in this analysis is exposure to other EDCs such as phthalates, polyfluoroalkyl substances, and polychlorinated biphenyls. These chemicals are known to interfere with thyroid hormone regulation and may have compounded effects when present alongside BPA exposure [5,6,17]. Future studies should consider a broader EDCs to better assess the cumulative impact of environmental exposures on thyroid function.

## Conclusions

This study highlights the impact of BPA exposure in early childhood on thyroid function. We found that BPA exposure at ages 7–9 years is significantly associated with decreased free T3 levels in girls at 10–12 years, suggesting that early school-age exposure is a critical window for endocrine disruption. In contrast, exposure at 3–5 years showed no significant association with later thyroid function, emphasizing the importance of exposure timing. Among the two points, exposure close to adolescence can affect the endocrine system in puberty, which might be related to an increase in thyroid disorder in adolescents according to environmental effects.

These findings have significant public health implications, as thyroid hormones play a crucial role in metabolism, growth, and neurodevelopment. Disruptions in thyroid hormone function during childhood may have long-term consequences on metabolic health and cognitive development. The increasing prevalence of thyroid disorders in children and adolescents underscores the need for public health measures to reduce BPA exposure. Further longitudinal studies with larger sample sizes and comprehensive exposure assessments are needed to clarify the long-term effects of BPA and potential interactions with other EDCs. A long-term follow-up of the affected children with their endocrine profiles, growth and neurocognitive development should be considered.
